# Missing appointments by patients on antiretroviral therapy: Professional nurses’ perspective

**DOI:** 10.4102/curationis.v45i1.2213

**Published:** 2022-01-31

**Authors:** Mygirl P. Lowane, Rachel T. Lebese

**Affiliations:** 1 Department of Public Health, Sefako Makgatho Health Sciences University, Pretoria, South Africa; 2 Research Office, Faculty of Health Sciences, University of Venda, Thoyandou, South Africa

**Keywords:** people living with HIV, antiretroviral therapy, missing appointments, professional nurses, perspectives

## Abstract

**Background:**

Missed appointments for medicine pick-ups are regarded as a predictor of poor adherence, and should trigger immediate questions about issues that may affect follow-up visits to healthcare settings.

**Objectives:**

The study explored and described professional nurses’ perspectives about the factors that contribute to missing appointments by people living with Human Immunodeficiency Virus (PLWHIV) on antiretroviral therapy (ART) at Mopani and Vhembe district in Limpopo Province.

**Method:**

A qualitative explorative contextual approach was used for the study. Non-probability, purposive sampling was used to select 14 professional nurses who met the inclusion criteria. Data were collected through face-to-face unstructured interviews. One central question was asked and probing questions were based on the participants’ responses to the central question. Thematic analysis of the findings was carried out. Trustworthiness was ensured through intercoder agreement, audio recording, triangulation, bracketing, and member checking. Required permission, approval, and ethical clearance were also ensured.

**Results:**

Organisational health system and management of the healthcare facility were found to be the barriers that negatively impacted on the ability of the PLWHIV on ART to maintain clinic visits appointments. Lack of patient involvement, stereotyped appointment dates selection, poor patient-provider relationships, errors of recording appointment dates and long waiting times came up as sub-themes derived from the main theme.

**Conclusion:**

The results suggest that there is a need to increase and improve mutual trust in patient-provider relationships, improve nurses working conditions, develop proper booking systems and reduce clinic waiting hours.

## Introduction

Under the antiretroviral therapy (ART) programme, regular clinic attendance for human immunodeficiency virus (HIV) management such as medicine pick-ups, routine clinical assessments and laboratory investigations is important to achieve treatment success (Abdulrahman et al. 2019). Non-compliance to this programme triggers immediate questions about issues that may affect attendance to healthcare settings (NDoH 2010). Missed appointment is when a patient has missed the next follow-up appointment immediately a day after an appointment date was scheduled but is reported to the health facility before three months’ elapse (WHO 2016). Chamberlin et al. (2021) highlighted that patients missing original appointments, later voluntarily visit their designated healthcare facilities to collect medicines. It was identified that a common quality indicator for patients’ retention is when a patient has attended two or more HIV medical care visits within a minimum of 90 days (Pence et al. 2019).

Studies conducted on the reasons for non-adherence to treatment programmes by people living with human immunodeficiency virus (PLWHIV) on ART revealed that factors such as cost, transport, waiting time, stigma, family pressures, religious beliefs and illness were prominent (Miller et al. 2010). Vagiri et al. (2015) and Bukenya et al. (2019) found that busy work schedules, work commitments and forgetting the appointment date were some of the individual factors contributing to non-adherence to the ART programme. A study conducted at three centres in Tanzania, Botswana and Uganda reported that the reasons for missing clinic appointments for about 50% of PLWHIV on ART included long waiting hours at ART centres, poor doctor-patient relationships and fear of retribution for having missed a previously scheduled visit, where patients sometimes delayed going to the clinic (Sakyi et al. 2015; Sastry et al. 2015; Tabatabai et al. 2014).

Long waiting hours was also affirmed by a study conducted in South Africa where it was reported that PLWHIV on ART were dissatisfied and complaining about clinic waiting times which they consider too long. Thus, they sometimes delay going back to the clinics for antiretroviral (ARV) treatment refills (Dahab et al. 2008). Magadzire, Mathole and Ward (2017) highlighted that healthcare practitioners felt that some of the reasons for non-adherence to appointments experienced by patients were beyond the scope of health services. Abdulrahman et al. (2019), in their study conducted in Baltimore Maryland, posited that healthcare providers’ availability, attitude and communication styles were assumed to negatively influence on the scheduling of HIV clinic attendance. Healthcare workers are also faced with an increasing number of complex choices regarding patients’ participation in decision-making (Lam et al. 2016).

Non-attendance generates social costs related to unused staff time and ineffective use of resources (Dantas et al. 2018). The failure to keep scheduled clinic appointments has been identified as a global problem where thousands of PLWHIV on ART miss their appointments daily in healthcare settings (Alhamad 2013; Perron et al. 2010; Tan et al. 2017). Missed HIV medical care visits are associated with deleterious clinical outcomes, including virologic failure, thereby increasing morbidity and mortality (Kempf et al. 2010; Pence et al. 2019). Missing appointments interfere with the appropriate care for patients and is a waste of medical and administrative resources (Alhamad 2013). Missed clinic visits by PLWHIV on lifelong ART should be given attention because they can be later labelled as lost to follow-up (LTFU) (Etoori et al. 2020a). Hence, it is crucial to identify patients at high risk of future non-attendance to clinic scheduled appointments to prevent drop-out and to address the critical continuum gap in HIV care engagement (Pence et al. 2019).

Missing appointments creates problems for essentially all healthcare systems and significantly impacts the functioning of healthcare institutions because patients need to be traced and reintegrated into the healthcare system (Tweya et al. 2018). Preventing patients from missing appointments may be more cost-effective than tracking those who do not return. The PLWHIV on ART receive extensive individual counselling on each visit. Return dates are documented in a patient-retained cards at each visit. Despite these efforts, PLWHIV on ART still do not honour their scheduled appointments. The investigators noted with concern that currently, there are limited studies in South Arica evaluating the magnitude of missing appointments amongst PLWHIV on ART in the healthcare setting. However, Fox et al. (2018) highlighted that the South African National Department of Health (NDoH) has indicated that retention in care and adherence to ART in South Africa are suboptimal and pose a serious threat to the long-term success of the national HIV response. Driven by the need to address these challenges of missed appointments in South Africa, this study sought to explore professionals’ perspectives on the factors that contribute to missing appointments and to suggest interventions to improve adherence to appointments amongst PLWHIV on ART programme.

## Research methods and design

### Study design

A qualitative descriptive and contextual study was used to explore the perspectives of professional nurses regarding the factors that contribute to missing appointments by patients on ART. The method was identified as being appropriate for the nature and complexity of the research problem. The method was affordable and assisted the researcher to understand the phenomenon under study from the participants’ perspectives.

### Study setting

This study was conducted in community health centres (CHCs) of Vhembe and Mopani districts in Limpopo province. These districts were selected purposefully because of their high rates of PLWHIV on ART missing their appointments. Vhembe district is found in the northern side of Limpopo province neighbouring Zimbabwe, whereas, Mopani district is found in the eastern side neighbouring Mozambique. These districts are mainly rural with patches of small towns. Both Vhembe and Mopani districts have eight CHCs. Nine CHCs with a high volume of above 30% of PLWHIV on ART missing appointments were selected, where four CHCs were identified from Vhembe district and five from Mopani district.

### Study population and sampling strategy

The accessible population for the study consisted of professional nurses who are trained in Nurse Initiated Management of Antiretroviral Therapy (NIMART), who provide care for PLWHIV on ART programme. All participants had been trained on comprehensive care management and treatment (CCMT) support training of HIV-positive patients, whereas five had been trained in adherence counselling, and two had undergone training in the advanced clinical care of HIV-positive patients. These trainings are crucial for the management of PLWHIV on ART, as it addresses many issues regarding the ART programme, which also includes adherence to ART clinical appointments. Non-probability purposive sampling was used to select all NIMART trained professional nurses who had more than one year of working experience on ART services (De Vos et al. 2017). These professional nurses were able to elaborate on the factors related to missing appointments by PLWHIV on ART as they were providing care to them at the time of data collection. Only 16 professional nurses participated in the study across the nine selected CHCs. The size of the sample was determined by data saturation when no new information was coming up from subsequent participants after the 16th participant, and there was no substantial addition to codes and themes being developed (Creswell 2015).

### Data collection

Contact with the professional nurses was made through the clinic operational manager. The researcher thereafter contacted the participants to introduce self and explained them the project. The participants signed informed consent and the date, place and time of the interview were agreed upon (De Vos et al. 2017). The main researcher who was trained in the qualitative approach conducted one-on-one in-depth interviews with the participants in English. Data were collected using an unstructured interview guided by one central question which was: ‘Can you explain to me what in your view are factors that contribute to missing appointment by PLWHIV on ART in your health facility’. Probe, paraphrasing and follow up questions were used to deepen the discussion. Observations of non-verbal cues and field notes were taken to support the findings (De Vos et al. 2017). The interview question was tested by interviewing two NIMART professional nurses from the non-selected facilities, to ensure that the question is well understood by participants and yielded appropriate results. Results from the two test interviews formed part of the data that was analysed (De Vos et al. 2017). There were no modifications required to the research question.

Repeating or rephrasing questions during interviews enabled both the researcher and the participants to remain focused (De Vos et al. 2011). Open-ended probing questions and paraphrasing were used to deepen the discussion and to allow the participants to freely express their views about factors contributing to missing appointments by patients on ART (Creswell & Poth 2016; Cresswell et al. 2017). The participants’ interviews lasted for 40–50 min which facilitated the participants to candidly share their views.

Space for the interview was requested from the facility manager, as all participants opted to be interviewed in their respective facilities. During each interview, the researcher wrote notes and audio recorded the discussion with the participants’ permission.

### Trustworthiness

Trustworthiness was ensured through several strategies, which are credibility, dependability, transferability, and confirmability (Schwandt, Lincoln & Guba 2007). The investigator spent three months in the field speaking with the participants, developing relationships, rapport and learning phenomena of interest. Participants’ interview sessions were also extended to 40–50 min. Confirmability was ensured by making provision for data recording using a voice recorder and making field notes to ensure that the conclusions, interpretations and recommendations of the study were traceable to the sources. Transferability was ensured by purposively selecting participants who met the inclusion criteria and by a detailed description of methods and setting. After three months of data collection and development of transcripts, appointments were made with all the participants to verify if the data collected were the true reflection of their responses.

### Data analysis

Transcription was done verbatim within 24 h of conducting the interview when the researcher could easily recall the interview. Transcribed data and field notes including observational notes collected from 16 participants in both Vhembe and Mopani districts were reduced and organised to give meaning. An experienced qualitative independent data coder was used to analyse data separately from the researcher and thereafter held a consensus discussion meeting on their results. Data were analysed thematically which involved reading, coding and themes developed (Bryman 2016; Creswell 2013). Data saturation was confirmed by scrutinising all the 16 transcripts to identify if new codes and themes emerge, but the researchers found that all the quotes used in the participants’ transcripts were supporting the identified themes.

## Ethical considerations

Approval for conducting this study was granted by University of Venda Higher Degrees Committee and Research and Ethics Committee (SHS/17/PH/13/1603) and the Limpopo Department of Health Research Ethics Committee. Furthermore, permission to conduct the study was also sought from the two district executive managers. The participants signed informed consent after receiving information about the study from the researcher. Consent was also given concerning the use of audio records for the interviews. The participants were informed about their right to participate voluntarily and that they were free to withdraw at any time during the study. The information received from the participants was treated confidentially and the participants were also informed that the information gathered may be used for writing reports or publications, but their identity would be protected.

## Results

Fourteen participants of which nine were female and five were male with a mean age of between 20 years and 49 years participated in the study. One main theme was derived from data analysis which was the organisational health system and management of the healthcare facility as a widespread challenge on PLWHIV on ART. The following five sub-themes were derived from the main theme: (1) lack of patient involvement/engagement; (2) stereotyped appointment dates selection; (3) poor patient-provider relationships; (4) errors in recording patients’ return dates; (5) patients’ long waiting time at healthcare facilities.

Non-adherence to appointments was said to be a widespread challenge amongst PLWHIV on ART. The study found that although there might be patient-related factors, such as distance, forgetfulness and financial challenges, health service-related factors rank high according to the participants’ perspectives. Organisational health system and management of the healthcare facility were reported to be the factors that might cause the patients not to effectively utilise the healthcare services, thereby resulting in missing appointments. The participants strongly felt that these facility-related factors were the main factors contributing to patients’ missing appointments, hence the focus in this article.

### Subtheme one: Lack of patient involvement/engagement

The participants admitted that they involved ART patients to a limited extent when deciding on the date of the patients’ next appointment. This was said to be attributed to the fact that they are working alone in the unit with numerous patients. They explained how most of the time they have to rush through their daily work just to finish the work for the day. It was noted that the participants had also delegated their responsibilities to lay counsellors, with the belief that patients receive adequate counselling from lay counsellors. A participants stated the following:

‘I normally do most of the talking, teaching the patient about the side effects, dangers of missing a dose, practising safer sex and condom use, including family planning, while busy completing the clinical stationery. After that, I issue treatment and write the return date. Then the patients leave without being involved.’ (P7, female, 36 years old)

The study found that the professional nurses were not aware about the role of the patients during ART clinic visits and whether patients should be involved in making the decision regarding their care plan. Some of the comments were as follows:

‘Ah!!, I have never thought about that, I think it is because she was the one who is sick, and it is her responsibility to be there in the session to receive medication.’ (P5, female, 44 years old)

[*hesitated*], ‘Eh! Does she have a role? Yah! I think her role is to ask questions and to seek clarity where she does not understand.’ (P14, male, 34 years old)

However, one participant indicated that he sometimes engaged patients by asking questions to assess their readiness to take treatment and to know whether the scheduled date will be feasible for them. He responded as follows:

‘In most cases I find out from the patient the information received during adherence counselling, checking their understanding of their illness and assessing whether the patient is coping with his/her condition. I also allow the patient to suggest the date in which it will suit him/her [*patients*].’ (PN16, male, 33 years old)

### Subtheme two: Stereotyped appointment dates selection

The results indicated the use of specific criteria for selecting dates for patients to come back for assessment and refilling of treatment. The finding revealed that the dates were not given based on the patient availability, but according to the number of scripts and number of tablets inside the container. The following extract indicates how return dates were allocated to PLWHIV on ART:

‘Honestly!!, I have never involved the patients in choosing the return date. I only count 28 days on the calendar and inform the patient. … I am also guided by the number of tablets the patient still has [*in his/her container*].’ (P14, 34 years old)

Hence, they followed those standard operational procedures (SOPs) irrespective of the patient’s concerns or challenges. Hence, other participants also stated that they followed those SOPs irrespective of the patient’s concerns or challenges. One participant mentioned that she depends on the unpublished SOPs for the management of HIV patients on the ART programme developed by the provincial HIV team and non-departmental partners which stipulate that the patients should come back for scheduled appointments every fourth week:

‘If we ask patients when they think they might come back; it might be confusing; you will find that it contradicts what is written in the standard operational procedures [*SOPs*]. The SOPs stipulate that all patients should return to the facility for treatment refill every fourth week after the last treatment was issued.’ (P12, female, 47 years old)

The findings revealed that two participants were not showing any interest in considering an alternative day when the date might not suit the patients as indicated in the following:

‘No ways, the patients are not allowed to choose the return dates. That is the role of the nurse to give patients return dates. The patient must adhere to that day so that the visit will be captured in the TIER.Net system. The system will be affected if patients choose their appointment dates.’ (P13, female, 36 years old)

TIER.Net is an electronic patient monitoring system South Africa introduced for HIV patients which includes capturing scheduled visits (Etoori et al. 2020b). However, one participant indicated involving patients in the allocation of the return date. A different response provided by this participant is as follows:

‘I sometimes ask the patients if the day given is OK, but most patients say the date is fine. Others will say, it will depend on whether they will get time off from work. Most patients don’t dispute the given date, they just don’t honour the appointments. But those who have serious and understandable challenges, I do sometimes issue 2 months’ scripts.’ (P11, male, 49 years old)

### Subtheme three: Poor patient-provider relationships

Some participants reported that poor patient-provider relationships were observed as a potential factor contributing to the missing of appointments by PLWHIV on ART. The participants mentioned that some patients make nasty comments about the way the nurses are providing the service indicating that their services were slow. Thus, the participants highlighted that they are sometimes already angry when the patient enters the consulting room:

‘When the patient enters the consulting room I just document the information in the register and give the patient his/her medication without altering any words. Sometimes I even forget to write the return date.’ (P6, male, 30 years old)

The participants indicated that even though the patients did not comment much, one could pick up negative attitudes from their non-verbal gestures, which affects the relationship between the providers and the patients. These participants added that the patients who had poor relationships with the healthcare providers were the ones more likely to miss appointments, compared to patients who had positive relationships with the healthcare providers. Furthermore, the participants stated that the patients with good relationships, communicated well, which made it easy to arrange alternative dates.

### Subtheme four: Errors in recording patients’ return dates

The participants highlighted that the working environment such as shortages of staff, unavailability of guidelines and counselling rooms inhibited them from providing good quality services. Four participants complained that because they had to do much clerical work such as writing and recording, duplicating information in different registers and patients’ files, they sometimes commit errors when writing return dates:

‘The writing is too much. Sometimes I even forgot to write the return date on the patient’s … card.’ (PN13, female, 36 years old)

‘Due to a lot of writing and talking, I sometimes write a wrong return date in the file which does not match with the patient’s return card. At the end, it appears as if the patients have missed the appointment meanwhile is a clerical error.’ (P3, male, 28 years old)

Three participants mentioned that sometimes they give the patients dates that are on weekends and when they come for treatment collections, the patients are returned back by healthcare providers without being attended. One participant mentioned that she by mistake wrote incorrect month and when the patient came to collect the medication, was denied and was told to come on the month written on the card:

‘I have written that the patient should come in August instead of July. When the patient came she was turned back, told that it was not her date to collect medication.’ (P2, female, 27 years old)

### Subtheme five: Patients’ long waiting time at healthcare facilities

Long waiting hours at healthcare facilities affect not only the quality of service rendered to the patients but also the motivation of the PLWHIV on ART to continue with their care. Long queues and long waiting hours were identified by the participants as contributory factors for the missing appointments. The participating professional nurses indicated that some reasons for long waiting hours were beyond their control. They mentioned that long waiting times demotivate patients, as a result, some patients give up and return home without being attended to:

‘Other patients grumble and quarrel while in the queue indicating that the line is moving very slow. You know there is nothing that I can do because as a nurse I multi-task and I do everything in the health facility. Sometimes you notice that some patients have already left without being seen.’ (P9, female, 38 years old)

Other participants noticed that some patients changed dates deliberately and came on Mondays instead of Thursdays in order to avoid spending long hours in the clinic:

‘… that is why on Mondays the facility has a high volume of patients resulting in long queues and longer waiting times.’ (P4, female, 34 years old)

## Discussion

The study explored what professional nurses have to say about the factors contributing to missing appointments by PLWHIV on ART. In this study, the participants perceived the involvement of patients when setting an appointment as not necessary. The PLWHIV on ART should be involved in their care plan to receive their optimum cooperation and to improve their health outcomes. Non-involvement can demotivate the interaction which often leads to patients being treated as objects. A study by Dang et al. (2017) affirmed this belief as the researchers maintained that even stable ART patients need intermittent reassurance to control their condition. It has also been noted that although patients value healthcare providers’ knowledge and recommendations, they still want to be asked what they think about their treatment plans (Dang et al. 2017). It is therefore imperative for healthcare providers to engage patients during their care as outlined by Mgbere et al. (2017) who indicated that patient-provider engagement is complex and that healthcare providers need skills for this purpose.

Insufficient engagement when providing care to ART patients often leads to non-involvement of patients when setting updates for the next appointment. From the participants’ responses, it is evident that patients’ needs concerning setting up dates and times for follow-up treatment are decided by the professional nurses only. Mannion and Exworthy (2017) indicated that patients wanted their personal needs to be considered and suggested that healthcare delivery systems should customise services for each patient, instead of using the ‘one size fits all’ approach. Efforts to develop customised care must be designed around an understanding of what happens around the patients and must incorporate individual patients’ preferences by focusing on shared decision-making concerning appointments and self-management. There is a need to think about designing health delivery systems to fit the needs and expectations of individual patients because the patients’ decisions about their healthcare could result in more favourable treatment outcomes (Mannion & Exworthy 2017). A proper booking system may be the key to resolving such challenges. It will enable the healthcare provider to prioritise the services and assist in the fair distribution of workload and human resources.

The study revealed that professional nurses are faced with poor execution of quality services. Inadequate human resources raise some major concerns, especially in South Africa which has had the largest HIV epidemic in the world, causing all health facilities to work under pressure (Mburu & George 2017). Health personnel ends up attending to large numbers of patients daily to avoid backlogs, leading to emotional burnout, stress and frustration (Mburu & George 2017). Runciman and Walton (2007) indicated that there is a need to recognise and acknowledge that healthcare professionals are fallible and may commit errors when caring for patients in poor working conditions where resources are limited. Runciman and Walton (2007) concluded that the human condition cannot be changed, but the conditions under which human beings work need to be improved to meet the patients’ needs.

Long waiting hours at healthcare facilities affect not only the quality of service provided to the PLWHIV on ART but also discourage them to continue with their care. Long waiting times were said to be the main source of dissatisfaction for patients attending public healthcare facilities in South Africa. Most facilities are expected to cope with large numbers of patients, not only HIV-positive patients but all patients in need of care, most of whom have limited resources (Egbujie et al. 2018). Soremekun, Takayesu and Bohan (2011) stipulated that waiting time is a key component for determining patients’ satisfaction levels, and significant efforts are necessary to reduce waiting times and improve the efficiency of health services. The study in Tanzania by Azia, Mukumbang and Van Wyk (2016) found that waiting times reached a maximum of 10 h to access ARVs and contributed significantly to reducing clinic attendances, thereby affecting adherence to treatment. These authors urged for more focus on patients’ lack of confidence in healthcare providers as a major concern, because of its potential to negatively impact patients’ abilities to access ART in future.

On the other hand, healthcare providers are urged to come up with innovative ways for reducing long waiting hours. However, this was refuted by Jiang, Abouee-Mehrizi and Diao (2018) in their study who mentioned that appointment scheduling is mainly used to manage access to services by matching the existing resources with the most suitable demand. This resulted in most patients not honouring their booked times but coming at one time, causing huge demands on existing human resources and aggravating the long queues and long waiting hours. Soremekun et al. (2011) stated that there is a need to improve healthcare providers’ communication skills, enabling patients to understand the complexity of their medical history, the tests ordered, their involvement in their care and its processes while visiting the health facility. In this way, patients will understand that everyone has different healthcare service needs and it would be better to tolerate long queues and understand the potential benefits for their treatment outcomes.

Our results raise questions about the delivery of care to PLWHIV on ART who are affected by poor health service-related factors. For that reason, fear may explain the higher rate of poor adherence to appointments particularly if factors contributing to missed appointments by PLWHIV on ART are not resolved. If healthcare providers inquire about a patient’s concerns and engage them in decision-making, they might identify which patients are likely to not keep their appointments and intervene properly to prevent minimising the trend of patients missing appointments. The PLWHIV on ART might benefit from intervention to improve adherence to appointments whereby they will receive an explanation of what to expect in their care and why it is important for their health outcome. Therefore, shared decision-making with PLWHIV on ART should be facilitated to help in selecting reasonable clinical options, including involvement in selecting return dates.

## Conclusion

The qualitative study was conducted to explore the perspectives of professional nurses regarding the factors contributing to missed appointments by PLWHIV on ART. Organisational health system and management of the healthcare factors were found to be the factors that negatively impacted the ability of PLWHIV on ART to maintain clinic visits appointments. Rather than looking for issues that might apply to poor patient engagement, poor patient-provider relationships, errors in recording patients’ return dates and long waiting hours, there is also an urgent need to obtain detailed descriptions of the patients’ perspectives in order to gain a deeper understanding of the reasons for poor clinic attendance behaviour to formulate hypotheses for future research. The results of this study suggest that there is a need to increase and improve trustful patient-provider relationships, nurses working conditions, a proper booking system and reduced waiting hours. Innovative interventions incorporating the above-mentioned factors and tailored to the specific needs of PLWHIV on ART and healthcare providers are needed to address the multidimensional aspects of poor clinic attendance to improve patients’ retention to care.
